# Supporting the health of working women in midlife: co-designing and testing the acceptability of a digital exercise programme

**DOI:** 10.1186/s12905-025-04244-7

**Published:** 2026-01-05

**Authors:** Helen Humphreys, Katharine Platts, Nik Kudiersky, Ursula Ankeny, Robert Copeland, Anna Lowe

**Affiliations:** 1 Centre for Behavioural Science and Applied Psychology, Sheffield Hallam University, Collegiate Crescent, Sheffield, S10 2BP UK; 2 Advanced Wellbeing Research Centre, Sheffield Hallam University, Sheffield Olympic Park, Sheffield, S9 3TU UK; 3 Sheffield Hallam University, 153 Arundel Street, Lab4Living, Sheffield, S1 2NU UK

**Keywords:** Women’s health, Strength training, Menopause, Midlife, User-centred design, Acceptability, Healthy ageing, Workplace health, Digital health intervention.

## Abstract

**Background:**

Women in midlife (WiML) report a range of physical and psychological challenges, including the transition to perimenopause and menopause. Combined effects of work and life stressors alongside menopause symptoms in working women are reported to increase absenteeism, reduce productivity and work satisfaction and may result in women exiting the labour market earlier than intended. The aim of this study was to co-design and test a novel exercise programme delivered via smartphone app for working women in midlife. The findings aim to inform targeted support to facilitate exercise for working women in midlife, that could be offered virtually and at scale.

**Methods:**

A two-phase process was used to co-design and test a digital exercise programme for WiML, to be delivered via smartphone app. In phase one, participants (*n* = 12) joined four co-design workshops where collected data was mapped against the Behaviour Change Wheel (BCW). A tailored exercise programme was designed by an exercise physiologist and physiotherapist to meet user needs with a focus on increasing muscle strength. In phase two, a prototype app was developed to deliver the programme digitally. The app was tested for acceptability by a cohort of working women from the education and private sectors (*n* = 16) using the Theoretical Framework of Acceptability as a reference.

**Results:**

Creating opportunities, both physical and social, were identified as the strongest enablers for improving engagement with strength training exercises for WiML, while automatic motivation was identified as a barrier. The app-based exercise programme was found to be low effort to engage with, but affective attitude towards the app was neutral and perceived effectiveness of the programme was low.

**Conclusion:**

A digital intervention was co-designed and tested by WiML. Findings suggest that combining a flexible programme of aerobic exercise, strength training and pelvic floor exercise via a smartphone application may be an acceptable way to support the health and wellbeing of working women in midlife. Optimisation of the app usability and functionality are needed prior to assessments of effectiveness. Digital workplace interventions are a potentially viable way to address disparities in midlife health and to support healthy ageing in women.

**Supplementary Information:**

The online version contains supplementary material available at 10.1186/s12905-025-04244-7.

## Background

Women in midlife (WiML) experience a range of physical and psychological challenges, including the transition through perimenopause to menopause (the ‘menopausal transition’) [1]. During perimenopause, the ovarian hormonal profile is unpredictable. Production of hormones such as oestrogen and progesterone reduces, [2] causing the loss of skeletal muscle mass and bone density [3], and a wide range of symptoms affecting weight management, mood, cognition, temperature regulation, pelvic floor function and other body systems [4]. Post-menopause, concentrations of oestrogen and progesterone are low, increasing risk of osteoporosis and cardiovascular disease [39].

There is evidence that increasing physical activity levels can improve health and wellbeing during menopausal transition. Exercise interventions involving aerobic exercise and resistance training can lead to reductions in body fat, preserved or increased muscle mass, and enhanced muscle strength and endurance [5, 6]. Strength training and weight-bearing exercise interventions can increase or preserve bone density and thereby reduce the risk of osteoporosis in later life [7]. Increasing physical activity levels can also have beneficial effects on mood, including reduced depression and anxiety, as well as improvements in symptoms of menopausal transition and quality of life [8–10]. Pelvic floor dysfunction may first present or worsen during the menopausal transition due to changing hormone profiles. Evidence suggests that pelvic floor muscle training programmes can improve symptoms such as urinary continence [11, 12]. NICE guidance recommends preventative pelvic floor exercises for all women across the life course [40]. When symptoms are present, women should seek personalised support from a healthcare professional, in the absence of symptoms.

Population-level data shows clear disparities in physical activity participation levels between men and women across the life course [41]. Survey data from Women in Sport suggests that 30% of participants reported becoming less active since menopausal transition, but 84% of inactive women would like to become more active. [42]. The collective evidence highlights the importance that physical activity can have for optimising health and wellbeing during and beyond menopausal transition and presents a clear rationale for targeted support for women in midlife.

A recent study examined the proportions of men and women meeting aerobic and muscle strengthening exercise guidelines. Due to the lack of consensus on what constitutes adequate strength exercise, the authors defined strengthening activity according to three separate definitions. When applying the most stringent definition only 4.1% of women met the criteria, compared to 7.3% of men. [13]. This is a marked disparity when it is known that women are at greater risk of osteoporosis, and that strength training is known to be an effective intervention [14]. Frequently cited barriers to engagement in strength training include gender-based stigma, discouragement and negative comments, unavailability or inaccessibility of strength training facilities, inadequate knowledge or misconceptions, time constraints and lack of supervision or routine [15, 16].

For some women, biological and psychological challenges in midlife can be compounded by multiple employment and caring responsibilities at this life stage, including children and older relatives. For working women, the combined effects of work and life stressors alongside symptoms of menopausal transition have been reported to increase absenteeism, reduce productivity and work satisfaction, and may result in women exiting the labour market earlier than intended [17, 18]. Employers are encouraged to raise awareness within their organisations about the challenges of the menopausal transition and offer tailored support for women in midlife [19, 20], but there remains a lack of workplace health interventions targeting women.

Limited evidence exists about the acceptability and effectiveness of workplace physical activity interventions for menopausal women, particularly those involving strength training. There is however some evidence that digital interventions are an effective option for this population, and that digital platforms can facilitate access to information, support the creation of communities and provide health support and resources [21]. Acceptability is often tested at the pilot stage of an intervention to assess feasibility and probability of uptake, with examples found in the testing of digital workplace health interventions such as virtual reality [22], and technology for mental health improvement [23, 24]. Designing strength training interventions that are tailored for and acceptable to working women in midlife could aid retention of skilled and experienced employees whilst also supporting their physical and mental wellbeing needs.

## Aim of the study

The aim of this study was to co-design and test a novel exercise programme delivered via smartphone app for working women in midlife, with a view to providing targeted support in a way that could be offered virtually and potentially at scale for large cohorts of employees. For the purposes of this study, ‘midlife’ was defined as occurring between 40 and 64 years, whilst acknowledging that individual life events and experiences can influence the timing of this life stage as much as chronological age. The study was divided into two phases, (i) programme co-design and (ii) user acceptability testing. The objectives were:

- To understand the barriers and facilitators to engaging in exercise training, particularly strength training, for WiML.
- To explore needs and preferences associated with an intervention to facilitate regular exercise training, particularly strength training, for WiML.
- To build and test the acceptability of a prototype digital intervention to support working WiML to engage in exercise training.

Both phases of the study were approved by Sheffield Hallam University Research Ethics Committee (REC IDs: 37050211 and 55467001).

## Methods

### Phase one – programme co-design

#### Participant recruitment

A convenience sampling method was used. Information about the study (phase one) was shared via organisational newsletters and social media at a large British university in 2022. Women expressing an interest were sent a participant information sheet via email and invited to discuss participation with the research team. Participants who volunteered to participate provided written, informed consent, and were sent joining instructions to attend virtual workshops.

#### Programme co-design workshops

Four virtual co-design workshops involving WiML were undertaken, each lasting two hours. Sessions were facilitated by a multidisciplinary group of researchers specialising in physical activity, behavioural science, human-centred design and co-design. During each co-design workshop, members of the research team populated online whiteboards with key discussion points. Handwritten notes and reflections were also captured, then reviewed and discussed after each workshop. Key points were used to inform the planning of subsequent workshops and were also fed back to participants regularly throughout the process. [See additional file 1].

The behaviour change wheel (BCW) [25] was used as a framework to underpin the co-design process. The BCW outlines a series of evidence-based steps designed to support the design of behaviour change interventions [see additional file 2]. Embedded within the framework is the COM-B model [26], which organises factors that can influence behaviour into three domains: capability (physical and psychological), opportunity (physical and social) and motivation (reflective and automatic). Co-design activities were iterative, building on each session, guided by the BCW.

#### Data analysis (phase 1)

Research team members met outside of the workshops to map emerging findings against each stage of the BCW. Following workshops one and two, themes from discussions and outputs from the workshops were deductively coded by two researchers (AL and HH) against the COM-B model, to generate an overview of barriers and enablers to strength training. Coding against the COM-B model was done jointly in person, so that agreement was reached through discussion. Images generated during workshops were translated into keywords and grouped along with other discussion notes into COM-B domains. Gaps in knowledge were identified to inform the planning of workshops three and four. Matrices provided within the BCW guide and behaviour change taxonomy were used to identify relevant intervention functions, behaviour change techniques and modes of delivery [see additional file 3].

#### Exercise programme development

Tailored exercise programmes within the app were designed by an exercise physiologist (NK) and physiotherapist (AL) to meet user needs identified in phase one (e.g., accessible, time-efficient).

Exercise programme content was informed by international physical activity guidelines, which recommend that adults engage in at least 150 min of moderate-intensity or 75 min of vigorous-intensity physical activity per week, in addition to muscle-strengthening activities on two or more days per week [35]. As the programme was designed to be pragmatic using bodyweight exercises, it was recognised that the stimulus provided was more consistent with strength-endurance training rather than maximal strength development. Nevertheless, bodyweight training has been shown to improve muscle strength in sedentary women [44]. This approach was intentionally chosen to prioritise accessibility, safety, and acceptability, particularly for participants with limited equipment or exercise experience, while still aligning with public health recommendations to engage major muscle groups regularly [35]. Pelvic floor exercises were included based on evidence of improved urinary continence outcomes [43, 45].

Exercise programmes were designed to range in difficulty (beginner, intermediate or advanced), frequency (1–7 days/week) and exercise type (strength, aerobic and/or pelvic floor). Participants had the option to review each exercise session and progress, regress, or maintain difficulty level for subsequent sessions. Progressions and regressions involved modifications to exercise session frequency (up to 5 per week), exercise intensity (e.g., speed of muscle contraction) and/or complexity (e.g., changes in body position, body mass distribution, or challenging balance). Table 1 summarises the content of each exercise session type.

**Table 1:** Exercise programme summary by exercise type

Strength-endurance training	• Designed to be completed without equipment, at home • Each session comprised two sets of four 1-minute bodyweight exercises, targeting large muscle groups. • Session duration up to 15 min including warm-up (3-min), cooldown (2-min), rest between sets (2-min). • Aim for at least two exercise sessions per week involving strength exercises.
Aerobic exercise	• Option to schedule up to seven aerobic exercise sessions per week. • 150 min of moderate intensity activity (feeling out of breath but still able to talk comfortably), or 75 min of vigorous-intensity activity (fast breathing and difficultly talking), or a combination. • Participants encouraged to reduce sedentary behaviour and incorporate aerobic exercise into daily activities such as brisk walking or stair climbing.
Pelvic floor exercise	• Option to schedule up to seven pelvic floor exercise sessions per week. • Exercise description included a range of pelvic floor muscle contraction techniques (fast and repetitive; slow and controlled). • 3–5 min duration for each pelvic floor exercise session.

### Phase two – app development and acceptability testing

#### App prototype development

A software company was commissioned to develop a prototype digital app, including the following features:

- Sign-up process including questions about contraindications, exercise training preferences and self-efficacy.
- Scheduling function, allowing users to drag and drop workouts to a 7-day calendar.
- Notifications to remind users to complete their scheduled workouts.
- Opportunities to longitudinally track health and wellbeing (e.g., current energy levels, sleep quality, and satisfaction with menopause symptom management).
- Placeholders for future insertion of expert-endorsed information articles focused on exercise training and women’s health.
- Exercise tutorials.

#### App prototype advisory group

A small advisory group of seven WiML, recruited via the University menopause support network, gave feedback on an early prototype of the app via wireframes. Characteristics of this group were not collected as they were not formal research participants. The advisory group were asked to navigate to the wireframes online and complete simple tasks such as creating a schedule or marking a training plan as complete. The group were informed that the images, content and functions needed refinement and that the app was going to be aimed at women who have little to no experience of strength training. Feedback was collated during an in-person session with the app developer, to inform refinement of the MVP (minimum viable product) for user acceptability testing. Group feedback centred around exercise adaptation for health conditions, social components, resource content suggestions, app layout and general ‘look and feel’. Screenshots of the prototype app can be found in additional file 4.

#### Participant recruitment

A convenience sampling method was used. Information about the study (phase two) was shared via internal communications channels at a large British university and a large private-sector business in March 2023. Women expressing an interest were sent information via email. Participants who volunteered to participate provided written, informed consent.

#### User acceptability testing

Participants completed a physical activity readiness questionnaire (PAR-Q) [27] to screen for contraindications to exercise, followed by instructions for downloading the prototype app and accessing the accompanying acceptability questionnaire. Participants were invited to download and test the prototype app for two weeks using the TestFlight app-testing platform. The test period was limited to two weeks due to project timelines. In the app, participants were asked brief questions about their current physical activity levels and exercise preferences. Their responses generated a tailored exercise programme using an automated decision tree built into the app.

In phase two, the Theoretical Framework of Acceptability (TFA) was utilised to assess the acceptability of the prototype app [28], using both quantitative and qualitative methods [29, 30]. The TFA conceptualises acceptability across seven domains: affective attitude, burden, ethicality, intervention coherence, opportunity costs, perceived effectiveness, and self-efficacy. Each domain was assessed using a 5-point Likert scale, with higher scores indicating greater acceptability. Items with negatively worded statements were reverse-scored. Following the two-week user testing period, participants completed an online questionnaire with questions about user demographics, perceived acceptability of the app, based on the TFA domains, and free-text qualitative feedback about their user experience (see additional file 5).

#### Data analysis (phase 2)

Descriptive statistics were used to describe and summarise data obtained from using the app and the follow-up questionnaire. Free text responses were thematically analysed, coded and organised using a priori themes from the TFA.

## Results

In phase one, 12 WiML participated in the co-design workshops. In phase 2, 24 women completed the physical activity readiness questionnaire (PAR-Q) [27], one was excluded, and of the remaining 23, only 16 downloaded the app. Participant characteristics are presented in Table 2.

**Table 2:** Participant characteristics

Characteristic	Phase one (n=12)	Phase two(n=16)
Age (years)		
40-44	1	0
45-54	6	12
55-64	5	4
Work status		
Full-time employed	7	14
Part-time employed	2	2
Unemployed	1	0
Retired	2	0
Ethnicity		
White British	9	15
Arab	2	-
Black or Black British - Caribbean	1	-
Black or Black British - African	-	1
Menopause status (self-identified)		
Pre-menopause	3	Not collected
Peri-menopause	4	
Post-menopause	2	
Don’t know	3	
Current experience of strength training (ST)		
Regular ST	2	Not collected
Occasional ST	6	
No regular ST	2	
Don’t know	2	

## Phase one – programme co-design

**Fig. 1:**
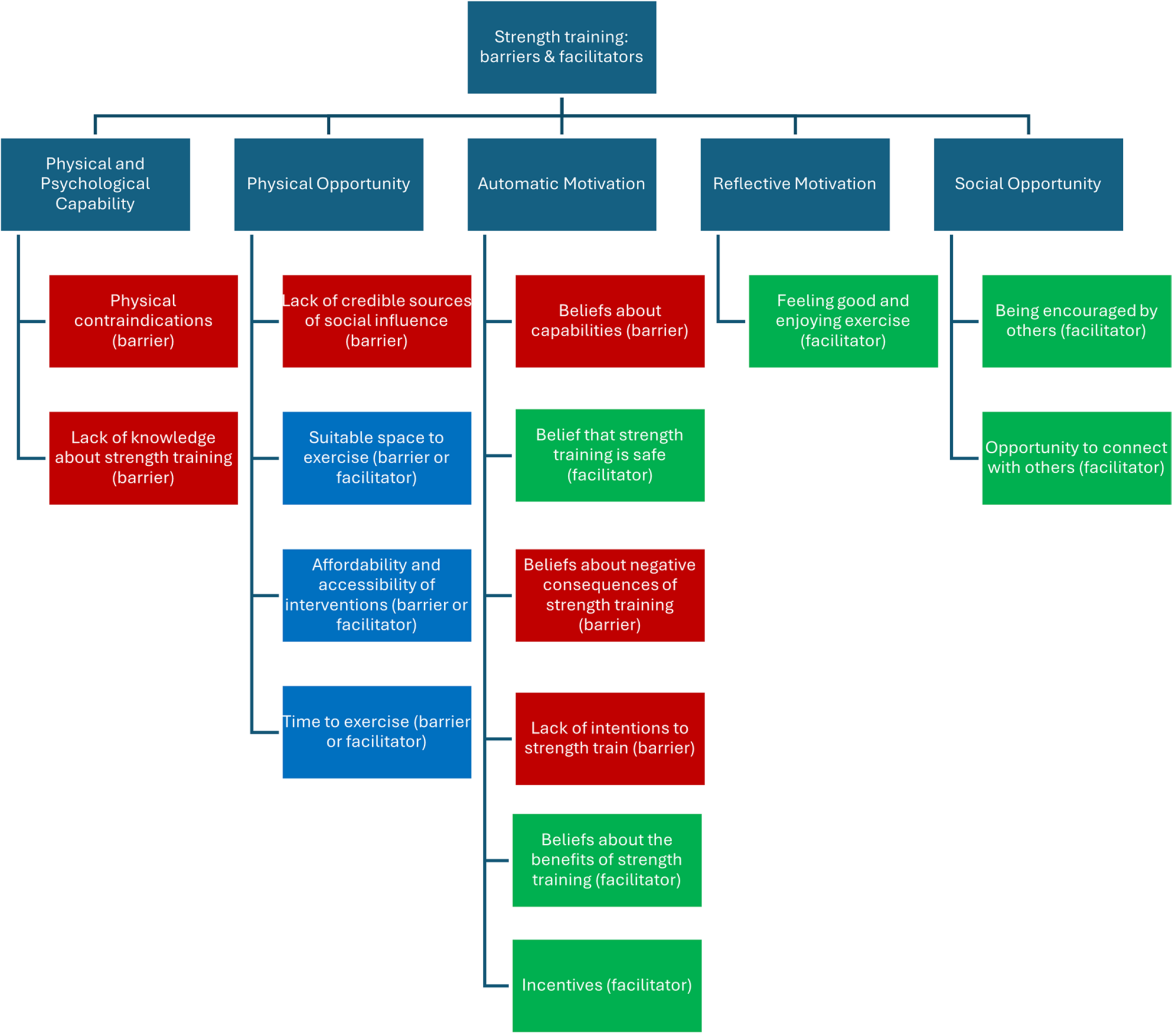
Key themes captured in co-design workshops organised by COM-B domain. Green = facilitator, blue = facilitator or barrier,  red = barrier

Key findings from the co-design workshops, organised by COM-B are presented in Fig. 1.

## Barriers and facilitators to strength training

Themes from the workshops are presented in narrative form below, organised by COM-B domains.

## Psychological capability

### Lack of knowledge about strength training (barrier)

WiML perceived strength training as complicated and reported that they lacked sufficient knowledge about how to start, what exercises to do, how to use equipment and whether it was possible to strength train without equipment. They described a common misperception that strength training should be undertaken in a gym environment and/or using traditional weighted equipment. There was little knowledge of guidelines or recommendations around the appropriate “dose”, i.e. how much and how often. Participants reported that they were not currently exposed to, nor able to easily access information about strength training.

## Physical capability

### Physical contraindications (barrier)

Some participants reported having physical conditions such as osteopenia or long-term pain which affected their mobility and presented a consideration for the suitability of some types of exercise.

## Physical opportunity

### Having a suitable space to carry out exercise including strength training (barrier or facilitator)

Many participants stated that they had never, and would never, access a gym. They suggested that home-based exercise was likely to be accessible for a wider range of WiML than a gym-based programme, although it was noted that some women may lack sufficient space or privacy in their home to exercise.

### Having the time to carry out exercise including strength training (barrier or facilitator)

Some participants cited a range of competing responsibilities including current employment, caring responsibilities, social relationships and many reported feeling time-poor. Conversely, others suggested that early retirement, reduced working hours, divorce and/or having older children meant they had more free time at this life stage. Regardless, flexibility was key; participants desired an intervention that could be completed anytime to suit individual need.

### Affordability and accessibility of exercise and strength training interventions (barrier or facilitator)

Cost was highlighted as a barrier for many WiML, and it was suggested that any intervention should be low-cost or free. A digital intervention was perceived as more accessible and less time-consuming than travelling to a face-to-face intervention, although it was noted that some WiML were digitally excluded.

## Social opportunity

### Lack of credible sources of social influence (barrier)

Participants discussed a mistrust of people or organisations ‘selling’ exercise products. Heavily promoted exercise interventions (e.g., by celebrities or social media algorithms) were sometimes deemed less trustworthy and less likely to be evidence-based. Local, familiar healthcare professionals (e.g., GPs) were considered a trusted source of information but most participants reported limited contact with these professionals.

### Opportunity to connect with others through exercise and strength training (facilitator)

Many participants predicted that they would respond positively to peer support from other WiML and regarded this as a key facilitator for ongoing engagement with an intervention. Some would value an intervention that could involve their family e.g., exercises they could do with grandchildren.

### Being encouraged by other WIML (facilitator)

Participants said that other WiML were their most trusted influences and would be encouraged to take up an intervention through positive case studies involving other women. Relatability was important, for example seeing women like them in terms of social class, local dialect, ethnicity and/or body shape.

## Reflective motivation

### Beliefs about capabilities (barrier)

Participants who did not routinely engage in structured exercise lacked confidence in their ability to undertake strength training. Physical considerations such as pelvic floor health and symptoms of menopausal transition such as anxiety, hot flushes and weight gain negatively impacted self-efficacy.

### Beliefs that engaging in strength training would have positive consequences (facilitator)

Positive beliefs about strength training benefits described by WiML included: increased psychological health and resilience; increased time for oneself; taking control and exercising freedom; enhancing other exercise abilities (e.g., being able to run further); healthy ageing (e.g., delaying the onset of age-related physical health problems). Some participants suggested that strength training might improve emotional resilience.

### Beliefs that engaging in strength training would have negative consequences (barrier)

Negative beliefs about the consequences of strength training were: potential for physical injury or exacerbation of existing conditions (e.g., osteopenia); too time-consuming; and a perception that strength training could potentially cause ‘bulking up’.

### Linking strength training to personal beliefs and values (facilitator)

Important values for WiML included: prioritising self-care; looking positively to the future; exercising freedom; building confidence (e.g., by trying new things); using personal time constructively; being a good carer. WiML suggested that linking strength training to these values could increase motivation to engage with an intervention.

### Incentives (facilitator)

Some participants suggested that they would respond positively to incentives (e.g., free coffees at work, charity donations) in exchange for adherence to an exercise intervention.

### Lack of intentions to initiate strength training (barrier)

Participants reported that although they weren’t directly opposed to engaging in strength training, they were also not proactively seeking it out. They suggested that intervention providers should provide information, incentives and motives for WiML to develop those intentions.

### Belief that a strength training intervention is safe and evidence-based (facilitator)

Given concerns highlighted around injury, participants suggested a need for reassurance about safety. Beliefs in “expert advice” had been challenged for some during COVID-19 and thus clear consensus from relevant “experts” was important.

## Automatic motivation

### Feeling good and enjoying exercise (facilitator)

Participants engaged in lively debate about the extent to which any exercise intervention could or should be “fun”. Some participants reported previously maintaining exercise behaviour despite not necessarily deriving pleasure from it. However, there was consensus that motivation would be higher and engagement easier if an intervention was perceived and experienced as enjoyable.

## Mode of delivery and intervention content

Based on the need for on-demand, flexible provision that considered women’s concerns and experiences of symptoms related to menopausal transition (such as pelvic floor health), co-design participants’ views suggested that there would be demand for a digital intervention to support strength training, that also incorporated aerobic and pelvic floor exercise. Mapping intervention functions and behaviour change techniques highlighted the importance of education around the ‘why’ and ‘how’ of exercise training. The proposed intervention needed to allow WiML to start at a level comfortable for them, tailored to fit around their current lifestyle, setting and tracking meaningful personal goals. Workshop discussions highlighted the importance of social influence and persuasion, indicating that an intervention could be usefully supported by employers but should be promoted and endorsed by other WiML. Imagery and language needed to be relatable and encouraging. A table mapping intervention functions and behaviour change techniques is available in additional file 3.

## Phase two – acceptability testing of prototype app

The acceptability questionnaire was completed by all 16 participants following the two-week app testing period. Participants were asked to self-report their adherence to the strength training plan as created for them by the app. In response to the question: “*how much of your plan did you complete?*”, one quarter of the participants did not adhere to their plan at all (*n* = 4), half the participants reported partial adherence (*n* = 8), and one quarter reported complete/mostly complete adherence (*n* = 4). Table 3 summarises scores from the acceptability questionnaire.

**Table 3:** Acceptability scores from post-intervention questionnaire (*n* = 16)

TFA Domain	Domain descriptor	Median (IQR)	Mode	Response descriptor (Median/Mode)
Affective attitude	Liked or disliked	3	2	No opinion/dislike
Burden	How much effort required	4 (4–4)	4	A little effort
Ethicality	Ethical concerns exist	5 (3.8–5.8)	5	Strongly disagree
Intervention coherence	I have clarity on how app supports WiML to engage in exercise	4 (4–4)	4	Agree
Opportunity costs	App interfered with other priorities	4 (3–4)	4	Disagree
Perceived effectiveness	Improved knowledge about the benefits of exercise	2 (2–3)	2	Disagree
	Improved exercise confidence	2 (2–3)	2	Disagree
	Improved physical ability to exercise	2 (2–3)	2	Disagree
	Improved mood	3 (2–3)	2	Disagree
	Reduced stress levels	2.5 (2–3)	2	Disagree
	Helped feel more relaxed	2.5 (2–3)	2	Disagree
	Felt physically stronger	3 (2–3)	2	Disagree
	Improved productivity at work	3 (2–3)	3	No opinion
Self-efficacy	Have confidence using app	4 (4–4)	4	Agree
	Have confidence doing exercise	4 (4–4)	4	Agree

## Affective attitude

Affective attitude refers to the feelings, or emotional responses, to the intervention. Overall, the affective attitude towards the app was ambivalence, which did not support a sense of enjoyment amongst participants, which was highlighted in phase one as important for motivation and engagement. Several participants highlighted that they valued the exercise tutorial information, including how to perform the exercise safely and effectively but many struggled with functionality issues within the app. One tester said they particularly appreciated the range of images portraying women of different body shapes, mirroring the comments made in phase one about the importance of *relatability* as a facilitator of exercise. A small number of testers said that they found the check-ins and notifications intrusive, but half of those who had used the app said they liked the notifications and reminders.

"I enjoyed the variation in the strength exercises between the weeks. The reminders also helped to keep me on track."

## Burden

Burden refers to the perceived effort required for participation. There was strong consensus that the prototype app was low burden, requiring required only ‘a little effort’ to use, meeting the need identified in phase one for *accessible* support. One user reported finding the workouts too long, particularly the warm-up and cool-down. Several participants found it difficult to stick to scheduled workouts due to other commitments or feeling too tired. They would have liked the option to reschedule workouts or break them down into shorter bouts, reflecting ‘lack of time’ as a barrier to exercise opportunity as identified in phase one.

"I needed to be able to break the workouts up into single exercises to do at various points throughout the day in order to fit it in. This wasn’t easily done."

## Ethicality

The ethicality domain generally refers to the extent to which the intervention matches with the participants value system. Users did not identify any negative moral or ethical consequences of using the app. Many testers believed the app to be a useful potential tool for employers to show support for WiML, with a focus on wellbeing as opposed to productivity. Workplace app provision, on a free or subsidised basis, would meet women’s needs for an intervention that was both *affordable* and *accessible*, as highlighted in phase one.

"…it’s a great start to promoting the impact of menopause, female health needs and finding ways to support women in the workplace better.

It shouldn’t be given in a paternalistic manner…It needs to be introduced from the point of view of ‘genuine’ support and not a solution to being more productive."

## Intervention coherence

Participants understood how the app worked and perceived that the app could help them engage in exercise training; critical components of the intervention coherence domain. Free text comments related to the functionality and usability of the prototype rather than its content or purpose. An exception was the pelvic floor exercise component. Some users suggested these could be improved by providing more detailed guidance on how to perform the exercises, which mirrored beliefs about personal capabilities and self-efficacy, as identified in phase one.

"More information [needed] on how to contract the pelvic floor muscles …when I attend for Pilates there are a variety of ways that the tutors (physios) explain how to do this which I think would be helpful in the app. The information sections are not always easy to navigate and flick through (a technical issue)."

## Opportunity costs

Opportunity costs are the benefits required to be sacrificed in order to participate in an intervention, and participants said that the app did not interfere with other life priorities. The main opportunity cost identified by participants related to time. In some cases, this was associated with challenges using the prototype rather than time for completion of the programme. Nevertheless, several participants highlighted time as an ongoing barrier to use, in the same way that phase one participants had identified ‘lack of time’ as a barrier to exercise engagement in general.

"I didn’t use it that regularly due to other commitments and largely forgetting to use it because of these, but it didn’t interfere with anything as I didn’t feel any pressure to use it."

## Perceived effectiveness

Participants were asked to what extent they agreed with eight statements relating to perceived effectiveness, which measured how likely they thought it would be to achieve its purpose. The perception of app effectiveness was low. This may be explained in part by differences in the extent of user engagement and the short testing timeframe of two weeks. Despite this, some users reported improved confidence, motivation and self-esteem.

"I probably would have to use the app more consistently over a longer period of time to have an answer to this."

"Improved my mental health as I was committed for two weeks to using the app. It spurred me on."

"I felt my self-esteem improved and my motivation to look after myself improved too."

"I am currently experiencing symptoms of menopause; however, I do not feel that the app necessarily improved or worsened these."

### Self-efficacy

Acceptability scores suggested participants felt confident in their ability to use and navigate the app and felt confident performing the recommended exercises, reflecting high levels of self-efficacy. Free-text responses conveyed a more varied user experience, however. For example, the scheduling feature was not adaptable enough for some, yet others thought scheduling was easy. Most feedback suggested that self-efficacy to perform the exercises was high, due to the quality of instructions accompanying each exercise, but a small number of users reported finding the exercises hard. Beliefs about personal capabilities related to exercise were noted as a barrier in phase one, which was clearly present in some phase two participants.

"Some of the exercises were too difficult for me. I also found the scheduling too rigid and would have liked to have had the option to change this."

## General feedback

### Supporting uptake and adherence

Participants were asked what should be done to promote uptake of the app and support adherence. Participants recommended improving app usability, more variation within the exercise sessions and more customisable goal setting and monitoring. Several suggested incentivisation, for example challenges or points for completing workouts, and a mechanism for users to connect and support one another. In the workplace, participants suggested that women could be encouraged to use the app if they were provided with safe spaces, time and social support:

"If this was an option within the workplace… then I would like to see options for women to have safe space to do this as a collective if wanted."

## Discussion

The primary aim of this study was to co-design and test the acceptability of a novel exercise programme delivered via smartphone app for working women in midlife, considering the specific needs, preferences and barriers to exercise engagement of this cohort. Barriers and facilitators to exercise participation were clearly elucidated in phase one in a user-centred co-design process using the COM-B behaviour change framework, with the strongest barriers to exercise identified as lack of physical and psychological capability (physical contraindications and lack of knowledge), and physical opportunity (lack of time). Key facilitators included high automotive motivation (personal beliefs about the benefits of exercise), and social opportunity (the connection and support of others). In phase two, an app-based exercise programme was developed comprising features designed to address the identified barriers and facilitators, and real-world acceptability testing was undertaken with a cohort of working women in midlife using the Theoretical Framework of Acceptability as a guide. Acceptability results were mixed. Participants generally had the confidence and understanding to use the app, but the emotional response to the programme was ambivalence and perceived effectiveness was low. The extent to which the app-based exercise programme (in phase two) was able to meet the identified needs of WiML (in phase one) may be a critical factor in understanding the mixed results achieved here.

### Capability to undertake exercise

Previous research has shown mobile exercise interventions via smartphone apps as potentially effective for increasing physical activity among midlife women with menopausal symptoms [31], which provided a strong basis for app development in this study. Poor knowledge and confidence however are known barriers to engagement with strength training and pelvic floor exercise for WiML [13, 15, 32], as are perceptions of reduced capability amongst menopausal women in relation to physical activity [33], both of which were identified as barriers in phase one of this study. In-app guidance and resources were provided, giving direction on how to perform strength exercises, and these were acceptable and useful for most participants; but some highlighted ongoing uncertainty about correctly performing pelvic floor exercises, meaning this barrier was not sufficiently addressed, despite positive findings for self-efficacy in phase two. Previous research has highlighted the importance and the difficulties of teaching women the importance of pelvic floor exercises [46], findings mirrored in this study. Further multi-media resources could be tested by women and included future app iterations, including for example 3D anatomical videos, talking heads from healthcare professionals, WiML describing their experience of learning how to do pelvic floor exercises, or external links to verified healthcare professionals signposted within the app. Findings from both phases highlight a clear need for support to build psychological capability and confidence in performing exercises correctly, in order to improve self-efficacy and underpin programme acceptability and sustainable behaviour change. Provision of exercise-related information *prior* to accessing a digital programme, particularly for women whose limited knowledge may be a barrier to initial uptake, may be a critical success factor in its effectiveness.

### Opportunity to undertake exercise

Gender-based stigma, poor body image and gym space accessibility are known barriers to strength training for WiML [34], although the most significant barrier may be competing demands of others during this life stage [16]. Digital interventions that offer on-demand, access anywhere exercise training plans, requiring no specialist equipment or access to a gym, enable WiML to train wherever is comfortable and convenient and maximise opportunity to exercise [31]. The app in this study included features designed to support behavioural regulation such as scheduling and notification features, yet adherence to the programme was low with only 25% of participants (*n* = 4) completing their entire exercise plan. This suggests that despite flexibility benefits and low effort required to participate, women lacked either the time or the motivation to fully engage and may explain why the perceived effectiveness of the programme was low.

It may be possible to address time-related barriers for WiML by breaking exercise training down into shorter doses. UK government guidance [35] acknowledges the challenges associated with setting a minimum dose for PA, given the broad spectrum of possible health outcomes and disease risk. Guideline amounts of aerobic activity can be accumulated through bouts of any length but guidance on muscle-strengthening activity is vague, suggesting this should be carried out twice per week but with little detail about what to do or for how long. Further research is needed to understand the most appropriate ‘dose’ of strength training to suit the health needs of WiML, and focusing on a minimum dose approach may be more helpful than debating ‘optimal dose’ in view of the extremely low rates of current exercise participation amongst this group.

### Social opportunity

Being encouraged by others, and the opportunity to connect with others, are identified in this study as core facilitators of exercise for women, and research suggests that social participation is critically important to exercise in midlife [47]. The very nature of digital exercise programmes mean they are less social than other types of group exercise interventions, highlighting the critical need for a social component in app-based options. Exercise app developers are encouraged to prioritise features that enhance social interaction, [48] and the lack of a strong social component in this app-based programme may have contributed to the general ambivalence of participants and poor adherence. Features such as group coaching, activity sharing, encouragement through ‘likes’ and social comparison (leaderboards and rankings) may all improve sense of connection and belonging, and improve motivation to engage [49].

### Motivation to exercise

Discussions with WiML in phase one highlighted that positive or negative beliefs about consequences could be a facilitator or barrier respectively to engaging in strength training, and previous research has found that appearing weak, silly or uncoordinated, fear of looking bulky, and judgement by friends exerts a powerful influence on women’s motivation to exercise [15]. Incentivisation was highlighted in phase one as a potentially useful form of motivation, and phase two feedback highlighted specific incentives, such as in-app challenges and reward points, that could encourage participation by adding gamified elements to exercise training.

It is in this space that employers could play an important role. Workplace physical activity challenges can be effective [36, 37] but more research is needed to understand how workplaces might effectively promote and incentivise uptake or engagement with interventions that might only be relevant to a relatively small, traditionally underserved part of a workforce [38]. Knowing the fundamental role of social connection and fun/enjoyment in sustained behaviour change, building these elements into exercise interventions offered to women at work, alongside incentives and rewards such as work-time exercise groups and social breaks, could be a powerful way to boost engagement and participation and improve workforce health.

This study addresses a clear evidence gap; it makes an important contribution to our knowledge of what works to support the physical activity and health of working women in midlife. Results indicate that there is a demand for digital interventions that combine aerobic, strength and pelvic floor exercise, but that critical elements such as skill development, confidence building and social support must be addressed in order for them to be engaging and effective at changing behaviour. While the app-based programme offered flexibility and the easy ‘on-demand’ accessibility highlighted as a preference of women in this study, it provided limited opportunities for in-depth or personalised support in skill development, or specialist adaptation in case of contraindications. The role of employers and the potential health opportunities that they can unlock for WiML is an important focus, for example in their ability to mitigate specific barriers including cost and time, and to provide ready networks of peers to provide social support. The innovative approach here combines co-design methods with acceptability testing, thus prioritising the views and experiences of end users in early-stage intervention development.

## Strengths and limitations

### Limitations

As noted throughout this paper, sample size in both phases were small and were drawn from two organisations. The convenience approach to sampling means that no generalisations can be made to wider groups and there is likely to be bias within the sample towards educated and motivated individuals who have the means and sufficient interest in the topic of strength training to participate. However, this approach was justified due to the exploratory nature of the research; broad applicability was not a primary aim of this study.

The online nature of the workshops may have impacted on participants ability and willingness to contribute due to issues of digital exclusion. However, the sample was drawn from two large institutions in which digital approaches are standard. The research team felt that on balance asking busy employees to travel to face-to-face events was more likely to form a barrier to engagement. The workshops themselves were not evaluated, so no conclusions can be drawn about participant experience or process effectiveness.

The timeframe for users to test the acceptability of the smartphone application was short at only two weeks. This was predominantly due to project timelines and resources and is noted as a particular limitation of the project. It is likely that it affected participants’ views on perceived effectiveness, a two-week period is not sufficient time to make meaningful physiological change due to exercise or meaningful behaviour change. A longer testing period is indicated for future studies.

### Directions for future research

Participants across both phases of this study emphasised the importance of social influence on exercise uptake and adherence. Other WiML were considered a trusted source of information and influence, who could potentially facilitate uptake and engagement through recommendation or buddying. The prototype app that was tested lacked a significant social component, this was highlighted by multiple participants as a potential area for improvement and is noted by the authors as an area for future exploration and development.

Participants reported that employers were potentially influential, particularly for their capacity to create opportunities to connect women within and across workplaces and promote exercise for women’s wellbeing. Participants in phase one highlighted cost as a potential barrier for WiML accessing an exercise intervention, in line with other reports in the wider literature [13]. They suggested the need for low cost or ideally no-cost interventions, which could be achieved via community-funded programmes or public health subsidies. For working women, an intervention paid for and made available by their employer is one evident way to mitigate this potential barrier. The current study did not assess acceptability from the perspective of employers, and this would be a crucial next step. Involving organisational representatives – such as those from HR or occupational health – could be a useful addition to the co-design and development process for future iterations of the app.

## Conclusion

To date, very little research has purposely explored the views of WiML regarding exercise and particularly strength training, yet the potential benefits of engaging in all forms of exercise for this population group are well-evidenced. The integration of exercise interventions for midlife and/or menopausal women into the workplace also remains relatively underexplored. The findings of this study suggest that a digital intervention combining a flexible programme of aerobic exercise, strength training and pelvic floor exercise may be an acceptable way to support the health and wellbeing of women in midlife in the workplace. A prototype app was acceptable in principle but significant improvements to usability and functionality are needed before it could be positively rated or assessed for effectiveness. More work is needed to understand how such interventions can be delivered and supported socially in the workplace. This remains a viable way to address disparities in midlife health and to support healthy ageing in women.

## Supplementary Information

Additional file 1: Co-Design Workshop Activities (Phase One).

Additional file 2: Co-Design Process based on Behaviour Change Wheel.

Additional file 3: Mapped Workshop Content defining Barriers, Facilitators, Proposed Programme Features, and Behaviour Change Techniques.

Additional file 4: Screenshots of prototype app.

Additional file 5: User Acceptability Questionnaire.

## Data Availability

The datasets used and/or analysed during the current study are available from the corresponding author upon request.

## Abbreviations

BCW: Behaviour Change Wheel
COM-B: Capability, Opportunity, Motivation-Behaviour
MVP: Minimum Viable Product
PAR-Q: Physical Activity Readiness Questionnaire
TFA: Theoretical Framework of Acceptability
ST: Strength training
WiML: Women in midlife
