# Supplementary material for: Supporting the health of working women in midlife: co-designing and testing the acceptability of a digital exercise programme
Source: BMC Womens Health. 2026 Jan 5;26:67. doi: 10.1186/s12905-025-04244-7 (PMC12869935; doi:10.1186/s12905-025-04244-7)
Supplement: Supplementary file 1 — Additional file 1: Co-Design Workshop Activities (Phase One). [file 12905_2025_4244_MOESM1_ESM.docx]

**Additional File 1: Co-Design Workshop Activities (Phase One)**

| **Workshop** | **Activity** | **Description** |
| --- | --- | --- |
| **Workshop One** | Image association | Discuss prompt statements with responses using image cards:  1) I feel strong when…  2) Strength to me looks like…  3) Midlife excites me because...  4) I am challenged by…  5) I am strong because… |
|  | Word association | Participants choose a word from list (or pick their own) to discuss. Prompts:1) I think strength training is…  2) Strength training is for… |
|  | Barriers and enablers | Developing a chart summarising barriers and enablers to strength training in midlife, using online whiteboard. Mood boards from previous image/word association activities to stimulate discussion. |
|  | Guiding principles for an intervention | Open discussion about ‘Guiding Principles’ for interventions in midlife. Prompt: Exercise interventions in midlife should be … |
|  | Brainstorming intervention ideas | Creative warmup activities followed by small group brainstorming ideas. Prompt: What might help women engage in regular muscle strength training? |
| **Workshop Two** | Draft guiding principles | Review, discuss and agree draft guiding principles for an exercise intervention developed from workshop one. Strength training for women in mid-life should be... (8 principles) |
|  | Reviewing intervention ideas from workshop one | Prompts:  1) What questions do you have about this idea?  2) Why would it work/not work?  3) What do you like/dislike?  4) What is your favourite and why? |
| **Workshop Three** | Analogous inspiration | Facilitators present images of existing PA interventions targeting WiML. Small group breakout discussions. Prompts:  1) Do you have experience of these?  2) Would you use any? If not, why not?  3) What do you like/dislike about them? |
|  | Online mood board | Building a mood board using online whiteboard and words/images from earlier discussions. Prompt: What is a digital intervention?  Discussion about different types of ‘digital’ (websites, email, WhatsApp/SMS, videos). Rank your preferred ‘digital mediums’ and share back to the group |
|  | Exercise content planning | Presentation and discussion about what constitutes strength training. Reviewing CMO physical activity guidelines and other evidence for exercise training |
| **Workshop Four** | Personas | Introducing personas to prompt discussion about how different women might use and engage with a digital intervention. Small group discussions:  1) Would certain approaches suit different circumstances better?  2) Is there another approach not considered?  3) What would suit you and your lifestyle?  4) Which would be the most motivating and for whom? |
|  | Storyboards | Facilitators introduce digital intervention concept using storyboards. Record comments and reflection.  Prompt: How could this be advertised or promoted to WiML? |
|  | Content discussion | Review examples of exercise video content and discuss preferences. Discussion topics:  1) Format (who it is delivered by; type of instruction)  2) Programme duration and volume (how much would/could you do per week?)  3) What information do you need e.g., safety/technique, health benefits? |
|  | Behaviour change discussion | Discuss benefits and drawbacks of self-monitoring, goal-setting, incentive/reward and progress tracker tools. Brainstorm other ideas for supporting capability, opportunity, motivation. Prompt:  If you used this intervention, what would be the most important/meaningful outcomes for you? Rank top 3. |
