# Supplementary material for: Supporting the health of working women in midlife: co-designing and testing the acceptability of a digital exercise programme
Source: BMC Womens Health. 2026 Jan 5;26:67. doi: 10.1186/s12905-025-04244-7 (PMC12869935; doi:10.1186/s12905-025-04244-7)
Supplement: Supplementary file 2 — Additional file 2: Co-Design Process based on Behaviour Change Wheel. [file 12905_2025_4244_MOESM2_ESM.docx]

**Additional File 2: Co-Design Process based on Behaviour Change Wheel**

**STEPS ONE AND TWO:**

Define the problem in behavioural terms and

Select the target behaviour

**STEPS THREE AND FOUR:**

Specify the target behaviour and identify what needs to change

**Codesign workshop 1**: Exploring what women in midlife think about physical activity and strength training and the barriers and facilitators to engaging with this behaviour.

**Codesign workshop 2**: Exploring ideas about how to address barriers and facilitators and strengths and limitations of different intervention approaches

**Research team** undertakes analysis of workshop data–coding using COM-B

**STEPS FIVE AND SIX:**

Identify Intervention functions and Policy categories

**Research team** uses APEASE criteria to select/reject intervention ideas (Acceptability, Practicability, Effectiveness, Affordability, Side-effects, Equity)

**STEPS SEVEN AND EIGHT:**

Identify BCTs and Content and Mode of Delivery

**Codesign workshops 3 and 4**: Exploring the potential acceptability of different modes of delivery, behaviour change techniques and intervention content.

**Research team** iteratively reviews data from workshops to generate list of components and features for digital intervention
