# Supplementary material for: Supporting the health of working women in midlife: co-designing and testing the acceptability of a digital exercise programme
Source: BMC Womens Health. 2026 Jan 5;26:67. doi: 10.1186/s12905-025-04244-7 (PMC12869935; doi:10.1186/s12905-025-04244-7)
Supplement: Supplementary file 3 — Additional file 3: Mapped Workshop Content defining Barriers, Facilitators, Proposed Programme Features, and Behaviour Change Techniques. [file 12905_2025_4244_MOESM3_ESM.docx]

**Additional file 3: Mapped Workshop Content defining Barriers, Facilitators, Proposed Programme Features, and Behaviour Change Techniques.**

| **Barrier or Facilitator according to COM-B** | **Features of the proposed intervention (Content/mode of delivery)** | **Intervention function according to BCW** | **Behaviour Change Techniques** |
| --- | --- | --- | --- |
| **Psychological Capability** | | | |
| Knowledge about exercise, strength and pelvic floor training – what and why | Videos or images showing how-to perform exercises  Educational content via information articles | Education  Training  Modelling | - Information about health consequences - Demonstration of the behaviour - Instruction on how to perform a behaviour - Action planning |
| **Physical Capability** | | | |
| Addressing physical contraindications | Screening for contraindications using Physical Activity Readiness Questionnaire (PAR-Q)  Customisable levels of difficulty | n/a | n/a |
| **Physical Opportunity** | | | |
| Suitable space to carry out exercise training | Digital platform (smartphone app). Bodyweight exercises not requiring equipment, feasible to do in home/limited space | n/a | n/a |
| Time to carry out strength training | Customisable frequency of sessions  Mechanism to set reminders/cues, scheduling feature  Limit workouts to short bouts | Enablement | - Problem solving - Action planning - Self-monitoring of behaviour |
| Affordability and accessibility | Digital platform delivered via workplaces | n/a | n/a |
| **Social Opportunity** | | | |
| Social influence / Connection with and encouragement from other WiML | Relatable images of women with realistic bodies and home surroundings  Promoted by other WiML and workplaces | Persuasion  Enablement  Modelling | - Credible source - Social support |
| **Reflective Motivation** | | | |
| Beliefs about lack of physical capability to take part | Relatable images of women with realistic bodies and home surroundings  Customisable levels of difficulty | Education  Persuasion  Modelling  Training | - Demonstration of the behaviour - Instruction on how to perform a behaviour - Behavioural practice/rehearsal |
| Beliefs about consequences of taking part | Educational content and information articles | Education  Training  Enablement  Persuasion | - Feedback on outcome(s) of the behaviour - Information about health consequences - Goal setting (behaviour) - Goal setting (outcome) - Review behaviour goal(s) - Review outcome goal(s) |
| Linking to personal beliefs and values | Simple, women-friendly language  Relatable images of women  Set and monitor goals and progress | Incentivisation  Persuasion | - Credible source - Feedback on outcome(s) of behaviour - Self-monitoring of behaviour |
| Incentives | Set and monitor goals and progress | Incentivisation | - Feedback on outcome(s) of behaviour - Self-monitoring of behaviour - Goal setting (behaviour) - Review behaviour goal(s) |
| Belief that intervention is safe and evidence-based | Educational content and information articles  Promoted via workplace and other WiML | Education  Persuasion | - Credible source - Information about health consequences |
| **Automatic Motivation** | | | |
| Feelings of enjoyment, fun | Relatable images of women with realistic bodies and home surroundings, women-friendly language and imagery.  Limit workouts to short bouts. Customisable programme - frequency, difficulty and combination of exercise types to suit preferences | n/a | n/a |
