# Supplementary material for: Supporting the health of working women in midlife: co-designing and testing the acceptability of a digital exercise programme
Source: BMC Womens Health. 2026 Jan 5;26:67. doi: 10.1186/s12905-025-04244-7 (PMC12869935; doi:10.1186/s12905-025-04244-7)
Supplement: Supplementary file 4 — Additional file 4: Screenshots of prototype app. [file 12905_2025_4244_MOESM4_ESM.docx]

Additional file 5: User Acceptability Questionnaire

Q1 From everyone in the Bia team thank you for volunteering to test out our new app. Please complete the questions below to let us know how you found it and anything you think we could improve.

Q54 How many strength training sessions per week did you have in your plan, and what difficulty level did you choose (light, moderate, hard, very hard)?

________________________________________________________________

Q53 How many aerobic exercise sessions per week did you have in your plan, and what difficulty level did you choose (light, moderate, hard, very hard)?

________________________________________________________________

Q52 How many pelvic floor sessions per week did you have in your plan?

________________________________________________________________

Q2 How much of your plan did you complete?

- All of it (1)
- Most of it (2)
- Some of it (3)
- None of it (4)

Q3 What do you think were the reasons why you did/did not complete the training plan?

________________________________________________________________

________________________________________________________________

________________________________________________________________

________________________________________________________________

________________________________________________________________

Q27 What other features of the app did you use?

|  | Used successfully (1) | Tried to use but had difficulties (2) | Did not use (3) |
| --- | --- | --- | --- |
| Scheduling workouts (1) |  |  |  |
| Reading information articles (2) |  |  |  |
| Push notifications (3) |  |  |  |
| Goal setting (4) |  |  |  |
| Progress monitoring (5) |  |  |  |

Q28 Please use this space to comment on any other features of the app which you used:

________________________________________________________________

________________________________________________________________

________________________________________________________________

________________________________________________________________

________________________________________________________________

End of Block: Feedback on app usage

Start of Block: Acceptability based on TFA

Q47 Did you like or dislike the Bia app?

- Strongly dislike (11)
- Dislike (12)
- No opinion (13)
- Like (14)
- Strongly like (15)

Q48 How much effort did it take to use the app?

- No effort at all (1)
- A little effort (2)
- No opinion (3)
- A lot of effort (4)
- Huge effort (5)

Q49 Did using the app have any negative moral or ethical consequences for you?

- Strongly disagree (1)
- Disagree (2)
- No opinion (3)
- Agree (4)
- Strongly agree (5)

Q6 Is there anyone that you think the app is unfairly biased towards and/or it is not appropriate for? (please give details)

________________________________________________________________

________________________________________________________________

________________________________________________________________

________________________________________________________________

________________________________________________________________

Q8 Please rate the following statements based on how you feel after using for two weeks:

|  | Strongly disagree (1) | Disagree (2) | No opinion (3) | Agree (4) | Strongly agree (5) |
| --- | --- | --- | --- | --- | --- |
| The app provides sufficient information about how to perform the exercises (1) |  |  |  |  |  |
| The app helped me to stick to my training plan (2) |  |  |  |  |  |
| The app provided trustworthy information about exercise for women in midlife (3) |  |  |  |  |  |
| Using the app has improved my knowledge about the benefits of exercise (4) |  |  |  |  |  |
| Using the app has made me more confident about exercising (5) |  |  |  |  |  |
| Using the app has improved my physical ability to exercise (6) |  |  |  |  |  |
| Using the app has improved my mood (7) |  |  |  |  |  |
| Using the app has reduced my stress levels (8) |  |  |  |  |  |
| Using the app has helped me feel more relaxed (9) |  |  |  |  |  |
| Using the app has made me feel physically stronger (10) |  |  |  |  |  |
| Using the app has improved my productivity at work (11) |  |  |  |  |  |

Q9 If you are currently experiencing perimenopause/menopause symptoms, did you feel that these were improved or worsened in any way by using the app? Please tell us below which symptoms were affected (or skip this question if you do not currently experience symptoms):

________________________________________________________________

________________________________________________________________

________________________________________________________________

________________________________________________________________

________________________________________________________________

Q50 Is it clear to you how the app can help women in midlife with their knowledge, skills, confidence, motivation and opportunities to engage in physical activity, strength training and balance?

- Strongly disagree (1)
- Disagree (2)
- No opinion (3)
- Agree (4)
- Strongly agree (5)

Q14 How confident do you feel about:

|  | Very unconfident (1) | Unconfident (2) | No opinion (3) | Confident (4) | Very confident (5) |
| --- | --- | --- | --- | --- | --- |
| Using and navigating the app (1) |  |  |  |  |  |
| Performing the exercises (2) |  |  |  |  |  |

Q51 Did using the app interfere with other priorities in your life?

- Strongly disagree (1)
- Disagree (2)
- No opinion (3)
- Agree (4)
- Strongly agree (5)

Q15 If you felt that the app interfered with other priorities, please tell us more about this:

________________________________________________________________

________________________________________________________________

________________________________________________________________

________________________________________________________________

________________________________________________________________

Q17 If the app was available beyond this 2-week testing period how likely would you be to continue using it?

- Very likely (1)
- Likely (2)
- No opinion (3)
- Unlikely (4)
- Very unlikely (5)

End of Block: Acceptability based on TFA

Start of Block: Improvements to the app

Q18 What do you think could be improved about the app?

________________________________________________________________

________________________________________________________________

________________________________________________________________

________________________________________________________________

________________________________________________________________

Q19 What did you like most about the app?

________________________________________________________________

________________________________________________________________

________________________________________________________________

________________________________________________________________

________________________________________________________________

Q20 Was there anything you strongly disliked about the app?

________________________________________________________________

________________________________________________________________

________________________________________________________________

________________________________________________________________

________________________________________________________________

Q21 What improvements or additional support would you recommend to encourage women to start using this app?

________________________________________________________________

________________________________________________________________

________________________________________________________________

________________________________________________________________

________________________________________________________________

Q22 What improvements or additional support would you recommend to help women to keep using this app over time?

________________________________________________________________

________________________________________________________________

________________________________________________________________

________________________________________________________________

________________________________________________________________

Q23 What considerations do you think organisations offering this app to their female employees should make about how it is offered/promoted as part of a workplace wellbeing package?

________________________________________________________________

________________________________________________________________

________________________________________________________________

________________________________________________________________

________________________________________________________________

Q24 Overall, how acceptable was the app to you?

- Completely unacceptable (1)
- Unacceptable (2)
- No opinion (3)
- Acceptable (4)
- Completely acceptable (5)

Q25 Any other feedback

________________________________________________________________

________________________________________________________________

________________________________________________________________

________________________________________________________________

________________________________________________________________

Q28 Sex (assigned at birth)

- Male (1)
- Female (2)

Q29 Age (Years)

________________________________________________________________

Q30 Postcode (first part e.g. S10)

________________________________________________________________

Q31 What is your ethnic group? A. Asian, Asian British, Asian Welsh

- Indian (1)
- Pakistani (2)
- Bangladeshi (3)
- Chinese (4)
- Any other Asian background (5)

Q32 Any other Asian background, please write

________________________________________________________________

Q33 B. Black, Black British, Black Welsh, Caribbean or African

- Caribbean (1)
- African (2)
- Other Black (3)

Q34 If other Black background, please write

________________________________________________________________

Q35 C. Mixed or Multiple

- White and Black African (1)
- White and Black Caribbean (2)
- White and Asian (3)
- Any other Mixed or Multiple background (4)

Q36 If other Mixed or Multiple background, please write

________________________________________________________________

Q37 D. White

- English, Welsh, Scottish, Northern Irish or British (1)
- Irish (2)
- Gypsy or Irish Traveller (3)
- Roma (4)
- Any other white background (5)

Q38 If other White background, please write

________________________________________________________________

Q39 E. Other ethnic group

- Arab (1)
- Any other ethnic group (2)

Q40 If you have ticked any other ethnic group, please write

________________________________________________________________

| Page Break |  |
| --- | --- |

Q41 Education and occupation What is the highest level of education that you received?

- Left school before 'O' levels or equivalent (GCSE, CSE) (1)
- 'O' level or equivalent (GCSE) (2)
- 'AS' or 'A' Level or equivalent (3)
- Undergraduate degree (4)
- Post-graduate degree (5)

Q45 Employment status (select more than one option if appropriate)

- Employed (1)
- Self-employed (2)
- Working full-time (3)
- Working part-time (4)
- Unemployed (5)
- Retired (6)
- Other (7)

Q43 Job role

________________________________________________________________

Q44 Job sector (industry)

________________________________________________________________

End of Block: Improvements to the app
