# Supplementary figures and images for: Supporting the health of working women in midlife: co-designing and testing the acceptability of a digital exercise programme
Source: BMC Womens Health. 2026 Jan 5;26:67. doi: 10.1186/s12905-025-04244-7 (PMC12869935; doi:10.1186/s12905-025-04244-7)

Additional file 4: Screenshots of the prototype app

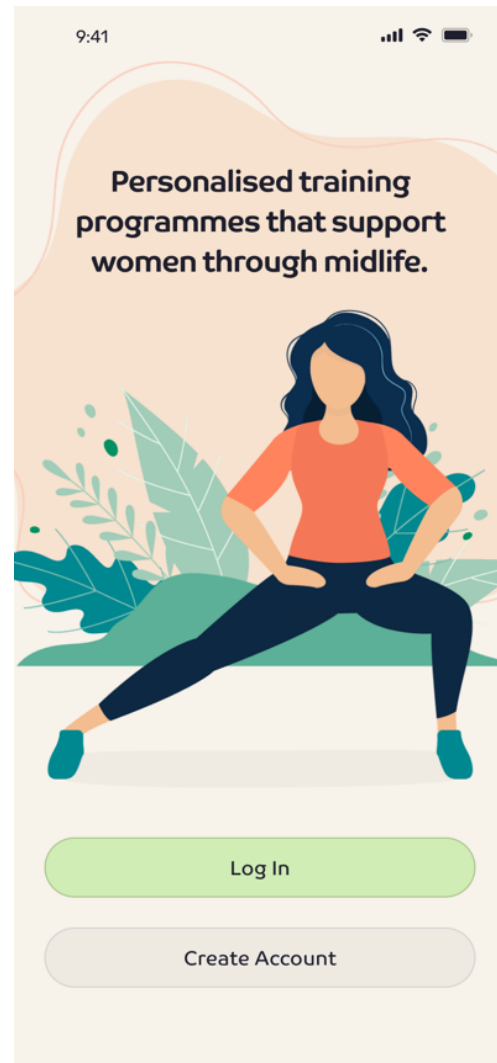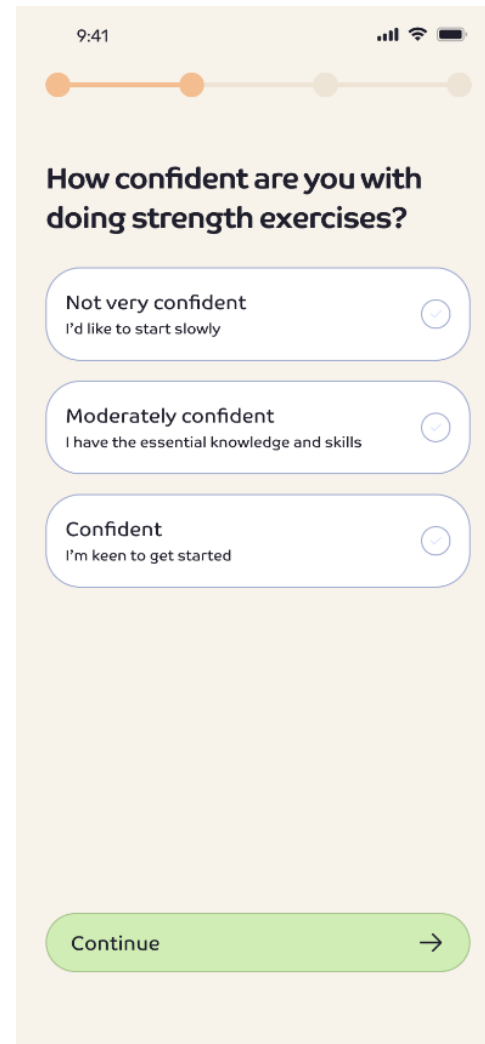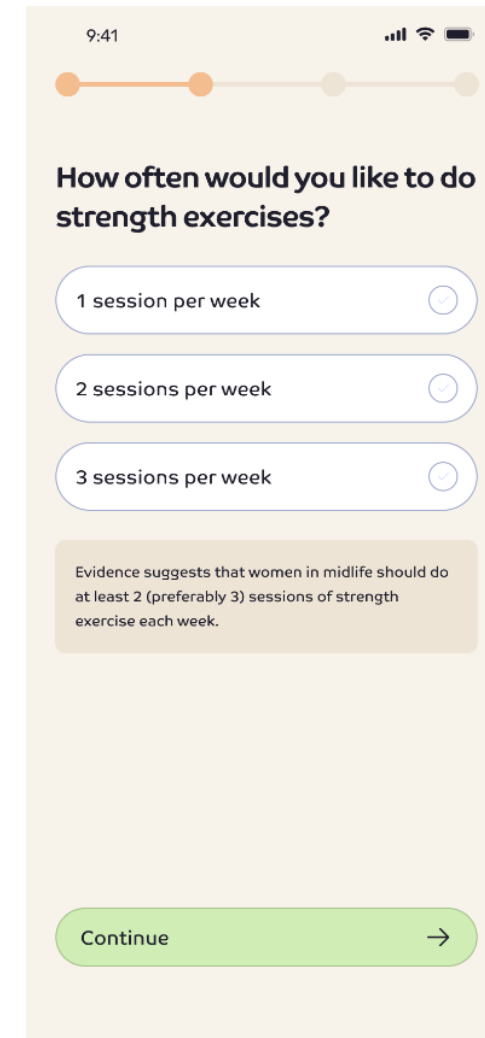

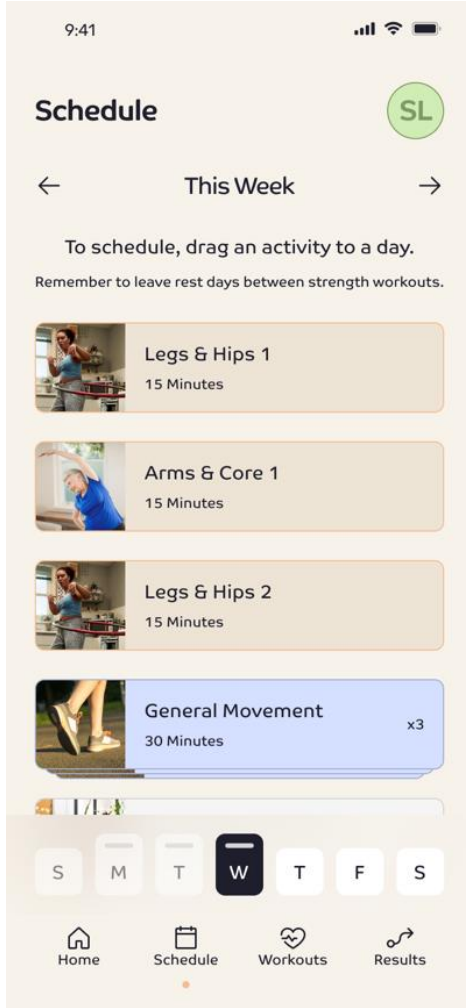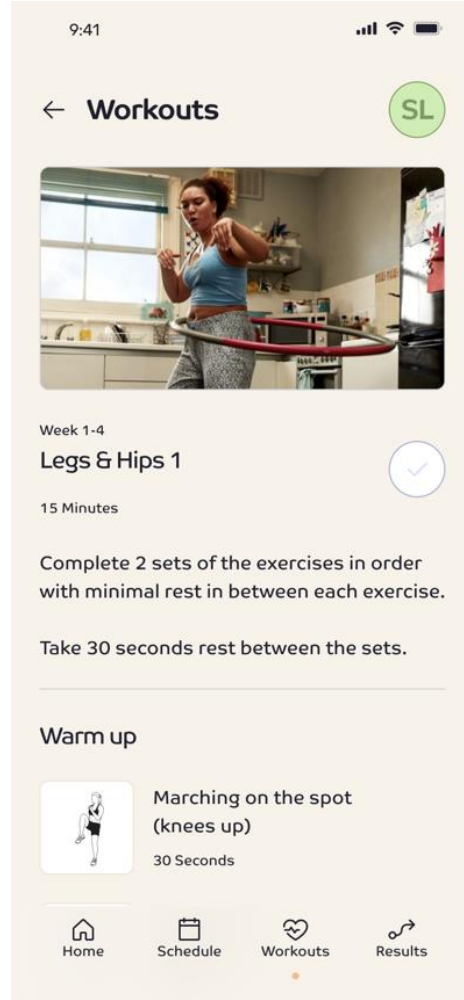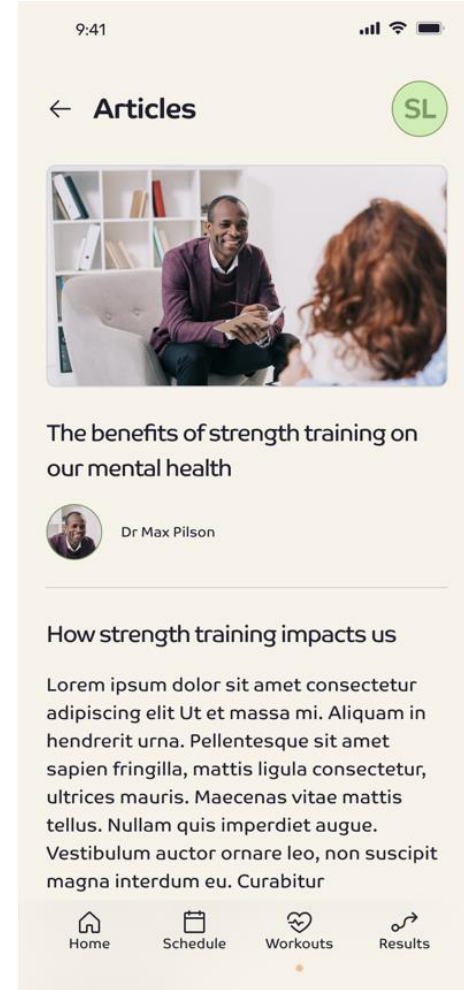

Supplement: Supplementary file 5 — Additional file 5: User Acceptability Questionnaire. [file 12905_2025_4244_MOESM5_ESM.pdf]
